# Tree Canopy Cover Is Best Associated with Perceptions of Greenspace: A Short Communication

**DOI:** 10.3390/ijerph17186501

**Published:** 2020-09-07

**Authors:** Soumya Mazumdar, Alison Dunshea, Shanley Chong, Bin Jalaludin

**Affiliations:** 1 South Western Sydney Clinical School, University of New South Wales Medicine, Liverpool, NSW 2170, Australia; Shanley.Chong@health.nsw.gov.au; 2 Population Health, South Western Sydney Local Health District, Liverpool, NSW 2170, Australia; Alison.Dunshea@health.nsw.gov.au (A.D.); Bin.Jalaludin@health.nsw.gov.au (B.J.); 3 School of Public Health and Community Medicine, University of New South Wales Medicine, Kensington, NSW 2052, Australia

**Keywords:** tree canopy, greenspace, perceived greenspace, objective greenspace, geographic information systems, buffers

## Abstract

A growing literature has supported a relationship between greenspace and health. Various greenspace metrics exist; some are based on subjective measures while others are based on an objective assessment of the landscape. While subjective measures may better reflect individual feelings about surrounding greenspace and the resulting positive benefits thereof, they are expensive and difficult to collect. In contrast, objective measures can be derived with relative ease, in a timely fashion, and for large regions and populations. While there have been some attempts to compare objective and subjective measures of greenspace, what is lacking is a comprehensive assessment of a wide range of greenspace metrics against subjective measures of greenspace. We performed such an assessment using a set of three objective greenspace metrics and a survey of residents in Liverpool, New South Wales, Australia. Our study supported existing findings in that overall, there is very little agreement between perceived and objective greenspace metrics. We also found that tree canopy in 10 min walking buffers around residences was the objective greenspace measure in best agreement with perceived greenspace.

## 1. Introduction

A large body of evidence has pointed towards a relationship between greenspace and physical or psychological health [1,2,3]. A number of metrics are used to quantify greenspace [4,5]. These metrics can be broadly categorized into three sets. The first set are instruments that measure an individual’s subjective experience of greenspace. This could either be a first person perception about greenspace, or it could be a third person/assessor generated metric of greenspace in specific neighborhoods. The second set includes objective Geographic Information System (GIS) based measures of greenspace, which include satellite-based metrics, such as Normalized Differential Vegetative Index (NDVI), and generally represent a bird’s eye view perspective [6]. The third set of methods attempt to objectively recreate the “on-ground” experience of the subject, using Google Street View (GSV) or similar tools to audit visible greenspace. While the first two method-sets of greenspace measurement are the most commonly used, GSV-based methods are being applied more often [7,8]. GSV-based methods attempt to reengineer the subject’s greenspace experience and have been shown to have stronger relationships with health outcomes than GIS-based methods [8]. Nevertheless, GIS-based methods and subjective assessments remain popular, particularly where a large amount of greenspace has to be assessed in a short period of time, as GIS-based measures can be quickly and cheaply generated for large areas with relative ease.

GIS-based measures of greenspace are comprised of two components. The first component is the spatial unit, buffer, or geographic container in which an individual’s greenspace experience manifests. The second component is the metric or construct that is used to measure greenspace within this container and includes measures such as percent parks or average NDVI. Metrics can be conceptualized and operationalized in a myriad of ways [6], depending on the construct being measured. For example, while a 100 m buffer around a residence is a good measure of perceived greenness around the home, the amount of greenspace within a ten minute walk-buffer measures greenspace within the resident’s walkable activity area. What is measured in these buffers is also important. While NDVI measures overall “greenness”, discretely marked green zones such as parks may represent public open spaces. The amount of tree canopy in such buffers has recently been shown to be more salient to health than other forms of greenspace [1]. Finally, even GSV-based metrics may have to be based on an optimal buffer size within which green exposure is measured.

Perceived measures of greenspace are often considered a “gold standard” for other measures to be compared against; one reason for this is that these measures often show the strongest association with health outcomes [5,9,10]. The different combinations of GIS buffer sizes, shapes, and greenspace metrics within buffers correlate differently with perceived greenspace measures [11,12]. We were interested in investigating how different objective measures of greenspace were associated with subjective measures of greenspace. While this research question has been addressed before, the general structure of existing analyses has addressed how a single objective greenspace measure in a specific GIS container compares with one or more perceived measure of greenspace [6,9,13]. Of the three research studies that addressed this question [6,9,13], one investigated perceived greenery, a GSV related measure, and green cover in 212 m buffers in Xalapa, Mexico [13]. Of the other two, one study from Seattle, USA used NDVI in a walking-time-based network buffer as a basis for assessing the green experience around the residence [9]. The third study from Adelaide, Australia used NDVI within 400 m buffers, apparently to approximate a 10–15 min walking distance, around the residence and a large set of subjective greenspace metrics [6].

While, like the previous researchers, we were interested in investigating if subjective measures of greenspace were related to objective measures in the neighborhood around the residence accessible by walking, we were also interested in examining the lived greenspace experience around residences, and how this related to objective greenspace/greenness measures. In addition, we were interested in moving beyond NDVI, and assessing other measures of greenspace. Note that previously, many researchers have attempted to investigate how relationships between various health outcomes and objective measures of greenness/greenspace differed from the relationships with subjective measures [5]. Our interest, however, was in assessing the direct relationship between objective and subjective measures of greenspace/greenness. Thus, we examined two different GIS containers, (i) a 10 min walking-network buffer and (ii) a 100 m buffer around the residence, and three different greenspace measures in the buffers, namely (i) percent tree canopy cover, (ii) mean NDVI, and (iii) percent parks. We compared these measures with two subjective greenspace measures: (i) a statement on greenery in the local area, and (ii) a statement on tree canopy along footpaths. Our goal was to identify which GIS–container–greenspace combination provided the best association with subjective measures of greenspace.

## 2. Materials and Methods

### 2.1. Data

#### 2.1.1. Subjective Greenspace Data

Data on subjective perceptions of greenspace were from the Liverpool Health and Lifestyles survey conducted by Population Health, South Western Sydney Local Health District, in Liverpool, New South Wales (NSW), Australia. It was an online survey on a range of demographic and health topics of residents in the Liverpool Local Government Area (LGA) conducted in March–April 2016. The LGA of Liverpool, like many other councils in Western/South-Western Sydney, is home to a very large and diverse, multicultural population. While the survey response rate was around 5%, we tested the survey for representativeness and validity, and it was found to have a greater percentage of older females and respondents facing psychological distress (not shown), but otherwise having a similar profile as the Liverpool LGA. The overrepresentation of a somewhat older and sometimes female demographic has been previously reported in Australian surveys [14]. Comparing early survey respondents with late respondents showed no significant differences (please see Tables S1 and S2).

The two survey questions/statements on perceived greenspace were: “There is lots of greenery (trees, bushes, gardens) around my local area”, and “There is tree cover or canopy along the footpaths in my local area”. The statements were adapted from the validated and widely used Neighborhood Environment Walkability Scale [15,16]. The responses were on a Likert-like scale with four categories of responses: Strongly Agree, Agree, Disagree, and Strongly Disagree.

A total of 307 individuals answered the survey; however, 68 respondents had either missing or inaccurate location information leading to an intermediate dataset of 239 respondents geocoded to their exact residence locations.

Various demographic and other information (described in the Methods section) were collected in the survey and subsequently included in our models as covariates. However, 100 respondents were missing various covariates and were excluded. The resulting dataset of 139 respondents had a very similar profile as the 239 respondents (Table S1). The 100 m buffers (described next) for three respondents were outside the Liverpool LGA and were excluded. Thus, the final dataset comprised 139 respondents for analysis utilizing 10 min walking buffers (described next), and 136 respondents for analysis using 100 m buffers.

#### 2.1.2. Objective Greenspace Data

The tree canopy dataset was obtained from Pitney Bowes Australia (PBA), which resells data originally developed by the Public Sector Mapping Agency (PSMA). The data were created by PSMA by combining classified 2 m visible, near infrared imagery data with digital elevation models. All 2 m pixels in the image were either classified by us as either having tree canopy or not having tree canopy [17,18]. The PSMA tree canopy data have been utilized previously by Australian researchers [2]. Next, we derived NDVI by processing a LandSat-8, 30 m resolution image (dated 11 April, 2013, April being the month of the above survey) with published algorithms [6]. Data on parks were obtained from PBA (StreetPro) and included parks, forests, and reserves such as state forests. All of NDVI, PBA parks and tree canopy data have been used previously by researchers [2,9,19]. We also obtained NSW Street Network data from PBA.

### 2.2. Methods

People may experience greenspace around their residence in different ways. First, they may experience ambient greenspace around their homes and residences, without actually stepping out of their homes. Second, they may experience greenspace when walking to destinations from their homes. These two ways of experiencing greenspace guided our choice of the two discrete buffer sizes/types in these analyses. A recent systematic review on mental health and greenspace found that a quarter of studies utilized buffers between 100 m to 800 m, or census geographies [20]. These thresholds also influenced our choice of buffer sizes. The lower threshold of a circular buffer of 100 m around the residence is appropriate for evaluating the environment immediately surrounding the residence (henceforward called 100 m buffers) [21,22,23]. Similarly the upper threshold of 800 m approximates a 10 min network walking buffer around a respondent’s residence location (henceforward called 10 min walk-buffer) [9,24]. Most people who walk for utilitarian purposes or recreation from their residences tend to do so within a 10 min waking time, with 75% of all walking trips in the Sydney metropolitan area being within one kilometer from residence [25]. Thus, not surprisingly, policymakers in NSW are attempting to increase the proportion of homes in urban areas within 10 min walking time to greenspaces [26]. Furthermore, researchers from the United States have analyzed National Household Travel Survey data to report a median walking trip time of 10 min [27].

**Figure 1:**
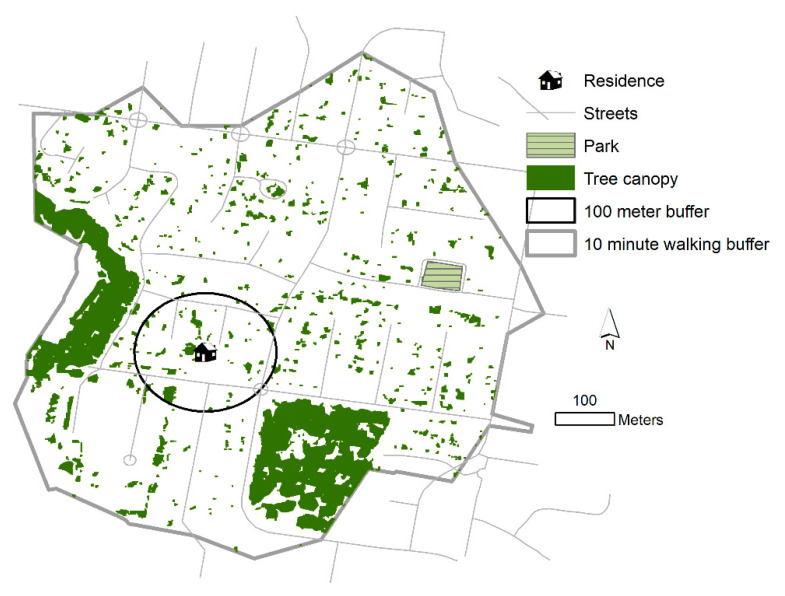
Sample residence with surrounding tree canopy, a park, and two types of buffers.

We evaluated the percentage area of the above buffers covered by parks and tree canopy. Mean NDVI in each buffer and the grand-mean of all NDVI in the study area were calculated. For analyses, the four subjective greenspace-related Likert scores were dichotomized/collapsed into agree and disagree categories. We dichotomized the percent park in buffer and percent tree canopy in buffer variables around the mean into binary 1/0 variables. If the mean NDVI in a buffer was greater than the grand mean, it was dichotomized into a variable with value of 1 (above average NDVI), or else 0 (below average NDVI). Figure 1 displays a sample residence with surrounding tree canopy, a park, and the two types of buffers.

We generated summary greenspace statistics for the two buffer types and then calculated raw correlations (Kendall’s Tau) between the dichotomized objective and subjective measures of greenspace. We predicted the odds of agreeing with the greenspace-related statements (dichotomized responses) as a function of the objective greenspace metrics and a set of confounders using logistic regression. A common set of confounders adjusted for in all models were age (categorized into three age groups (0 to 39 years, 40 to 59 years, and 60 to 79 years); sex (male, female); country of birth (Australian born, overseas born); and education (vocational certificate, diploma and university, or other tertiary institute degree or higher, versus high school or less). Since people who walk to and from their residences are more likely to be exposed to greenspace, especially along footpaths, we also adjusted for recreational walking (dichotomized at the median of twice a week) and utilitarian walking dichotomized at the median of 60 min/week in regressions predicting perceptions of tree canopy along footpaths. To infer the exact amount of additional variation (measured by a pseudo R-squared statistic) explained by the greenspace variable, each model was run with and without the greenspace variable. Thus, a total of twelve regressions were run (2 Buffer types × 3 Greenspace metrics) × 2 models. A standard logistic regression model was used, and Table S4 provides the model used and the list of confounders listed above. All statistical analyses were completed in R, version 3.6.0 [28], and all spatial analyses were implemented in ArcGIS 10.7 [29].

## 3. Results

The mean age of survey respondents was 43 years, with 24% male and 76% female. Seventy seven percent had completed a university or vocational degree/certificate. Forty three percent walked at least 60 min/week for utilitarian purposes, while 52% walked at least twice a week for recreation. Only 35% were born in Australia, reflecting a demographic composition closer to the Central Business District of Liverpool, where 31% were born in Australia. Table 1 shows the average amount of greenspace in the various buffers. Generally, 8–9% of both types of buffers had parks or tree canopy. Percent parks and tree canopy showed large variations (around 3–11%) in the buffers. NDVI ranged from around 0.3 to 0.4.

Correlations between the various greenspace metrics were generally low, except between mean NDVI and percent tree canopy, where it ranged between 0.3 to 0.4 (Table S3). Correlation between the two perceived greenspace metrics was also in this range at 0.35 (not shown). The metric combination that best explained perceived greenspace was percent tree canopy in 10 min walk-buffers. Both perceptions of overall greenspace and perceived footpath tree canopy were significantly associated with the percent of tree canopy in 10 min walk-buffers (Table 2). There was also a significant relationship between mean NDVI in 100 m buffers and perceptions about overall greenspace. This may be expected, given the relatively high correlation between mean NDVI and percent tree canopy in both GIS containers (Table S3). The model with the best explanatory power (9%) was the one modelling overall greenspace using percent tree canopy in 10 min walk-buffers. NDVI in 100 m buffers explained 4.3% of the perception of overall greenspace, which was the highest amount of variation explained by an objective greenspace metric that was also significant. Nevertheless, across all metrics, only a small proportion of the overall variation in perceived greenspace was explained by the objective greenspace measures (3–4%). None of the cofounders were significant, except for education, which was significant in four of the twelve models (not shown). In these four models, respondents who agreed or strongly agreed that there was tree cover or canopy along the footpaths in their local area were significantly less likely to have university/tertiary or vocational degrees/certificates (odds ratio ~0.4).

## 4. Discussion

An important finding of this study was that perceived greenspace was most strongly associated with percent tree canopy in 10 min walk-buffers, though the variation explained by this objective greenspace measure was small. The amount of tree canopy along and around walking routes from one’s home was important in not only affecting perceptions about greenness along footpaths, but also about overall greenspace in the local area. Greenness, measured by NDVI, within 100 m from residence, which is likely visible greenspace from home, was better correlated with perceived overall greenspace. Percent parks within any kind of buffers was not associated with perceived greenspace.

A number of recent studies have associated tree canopy cover with better mental health [30,31,32] and physical health [2,30]. One paper specifically found that within 1000 m buffers, tree canopy but not grass cover was related to significantly better health outcomes [31]. Another paper reported associations between various chronic health outcomes and tree canopy, but not with total greenspace [2]. Thus, tree canopy provides the best objective measure of greenspace and correlates best with perceived greenspace. A secondary observation was that NDVI within 100 m of residence was associated with perceptions about overall greenness. Indeed, NDVI within 100 m of residence has been validated as a measure of greenness in a separate study [10].

In the past, a handful of studies have attempted to disentangle the relationship between objective and subjective measures of greenspace. Two of these papers attempted to find the relationship between subjective measures of greenspace and a single objective greenspace measure (though operationalized differently in the two papers), as NDVI in walking buffers around the residence. Very low or no associations were reported between subjective and objective greenspace measures [6,9]. This has been construed to have resulted from the differences in the psychological construct measured by the subjective questions versus the objective measures [6]. It has also been shown that when subjective measures of greenspace were aggregated to larger geographies, they provided better agreement with objective measures. The authors attributed this to smoothing of individual level errors/variation with aggregation [33]. Our study shows that tree canopy-based measures could play an important role in aligning the objective and subjective constructs. For instance, GVI-based indices often categorize all “green pixels” as greenspace [34]. Perhaps separately identifying tree canopy objects in GVI-based indices could offer tighter associations with perceived greenness.

The three metrics used in this study measured three somewhat different aspects of greenspace/greenness. While NDVI measures “greenness”, tree canopy cover is a good measure of green coverage and shade. Finally, parks measure discrete organized greenspace. NDVI values are dependent on how green the given vegetation is, and the values in our study (0.3–0.4) were within range of Australian NDVI values [35]. While some measures were found to better measure perceived greenspace than others, the fact remains that even the best model was able to predict only 9% of the variation in perceived greenspace. This is expected, as GIS-based measures provide a “bird’s eye” perspective, which are appropriate for large-scale estimation of greenspace and not for exactly replicating the personal perceived greenspace experience for which “on ground” GSV-based methods have been developed.

There are a number of limitations of this study. First, the study was based on a relatively small survey, though even with small numbers, the effect sizes were large enough to reach statistical significance. Whether the tree canopy-related results reported in this study would have policy and practice implications would depend on replicating this study on larger samples, and further comparing and contrasting the differential effects of tree canopy on health [2,3]. Second, GSV indices could not be included in this study, and they remain an area of future research. Third, this study examined a set of two buffer sizes: 100 m and 10 min walking buffers. While our tree canopy-related findings were consistent across the two buffer sizes, it is not known what the results would have been if a larger set of buffer sizes was evaluated, and this also remains an area of future examination. Fourth, some of the differences in results in this study between the various objective greenness/greenspace measures could have resulted from the different resolutions of the datasets used. Thus, while the tree canopy data were at 2 m resolution, the NDVI data were at 30 m resolution. Fifth, the survey data had an overrepresentation of older females, which may have biased the results if this group had different patterns of greenspace perception compared to the general population. Finally, two specific subjective greenspace questions/statements were analyzed, and while the questions were standardized and validated questions, other questionnaires may have provided different associations.

This study has four broad implications. First, researchers and policymakers may need to refocus their energies on the location, quality, and quantity of tree canopy in addition to overall greenspace or other forms of greenspace. Second, researchers need to carefully consider which measure(s) of greenspace (either objective or subjective) are relevant for their study and not assume that these measures are necessarily related. Third, psychological constructs of perceived greenspace may be targeted towards measuring tree canopy in addition to overall greenspace. Finally, GSV and related metrics could be realigned to additionally measure tree canopy along with measures of total greenspace.

## 5. Conclusions

This study attempted to find the extent of agreement between different objective measures of greenspace and two subjective measures of greenspace. It found, in agreement with previous researchers, that there was poor agreement between objective and subjective measures of greenspace/greenness. The best agreement was found between perceived greenspace and tree canopy in 10 min walking buffers. This research supplements an increasingly large body of research underscoring the importance of tree canopy in greenspace-related benefits.

## Figures and Tables

**Table 1:** Percent of different metrics of greenspace in two different types of buffers. Figures in brackets show the 25th and 75th percentiles.

	100 m Buffer	10 min Walk-Buffer
Percent Tree Canopy	8.64 (3.86, 10.26)	9.67 (6.44, 11.49)
Mean NDVI ^1^	0.31 (0.28, 0.40)	0.37 (0.33, 0.43)
Percent Parks	8 (3.03, 10.97)	8.08 (3.08, 11.09)

^1^ NDVI stands for Normalized Difference Vegetation Index.

**Table 2:** Odds of agreeing or strongly agreeing with statements about perceived greenspace as a function of objective greenspace variables, with 95% confidence intervals in brackets. Odds ratios associated with the objective greenspace-related variable are shown from 12 models. The odds ratios associated with other covariates in these models are not shown.

	Tree Canopy		NDVI		Park Percent	
	**(1) 100 m Buffer**	**(2) 10 min Walk-Buffer**	**(3) 100 m Buffer**	**(4) 10 min Walk-Buffer**	**(5) 100 m Buffer**	**(6) 10 min Walk-Buffer**
***Perceived footpath tree canopy*** ^1^						
**Odds ratio**	1.17 (0.58, 2.38)	3.4 (1.62, 7.40) *	1.73 (0.83, 3.71)	1.2 (0.59, 2.51)	1.40 (0.68, 2.92)	1.73 (0.84, 3.65)
Overall variation explained by model ^2^	2.91	6.26	3.92	2.41	3.25	3.44
Variation explained by green variable ^2^	2.80	2.25	2.8	2.25	2.8	2.25
	**Tree Canopy**		**NDVI**		**Park Percent**	
	**(7) 100 m Buffer**	**(8) 10 min Walk-Buffer**	**(9) 100 m Buffer**	**(10) 10 min Walk-Buffer**	**(11) 100 m Buffer**	**(12) 10 min Walk-Buffer**
***Perceived overall greenspace*** ^3^						
**Odds ratio**	1.83 (0.91, 3.75)	2.77 (1.32, 6.01) *	2.12 (1.01, 4.59) *	2.12 (0.83, 5.67)	1.54 (0.75, 3.21)	1.53 (0.62, 3.91)
Overall variation explained by model	5.80	9.02	6.37	6.44	5.02	5.22
Variation explained by green variable	4.31	3.22	4.31	4.59	4.31	4.59

NOTE: Models have been adjusted for age, sex, country of birth, and education. The Walkable greenspace models (10 min walk-buffer) have been adjusted for minutes of utilitarian walking and recreation walking. NDVI stands for Normalized Difference Vegetation Index. * Odds ratios are significant (*p* < 0.05). ^1^ Outcome based on survey question/statement: “There is tree cover or canopy along the footpaths in my local area”. ^2^ Pseudo R squared statistic. ^3^ Outcome based on survey question/statement: “There is lots of greenery (trees, bushes, gardens) around my local area”.

## Supplementary Materials

The following are available online at https://www.mdpi.com/1660-4601/17/18/6501/s1, Table S1: Percentage distributions by Age and Sex in the survey compared to Liverpool LGA, Table S2: Comparing early responders with late responders, Table S3: Correlations (Kendall’s Tau) between different objective greenspace metrics, Table S4: Logistic Regression Model.
