# Supplementary material for: Tree Canopy Cover Is Best Associated with Perceptions of Greenspace: A Short Communication
Source: Int J Environ Res Public Health. 2020 Sep 7;17(18):6501. doi: 10.3390/ijerph17186501 (PMC7558589; doi:10.3390/ijerph17186501)
Supplement: Supplementary file 1 [file ijerph-17-06501-s001.pdf]

Supplementary material: Survey Validity

The Liverpool Health and Wellbeing survey design utilized a mail in prompting letter to all residents in the Liverpool Central Business District with a link to an online survey to answer an online survey. The survey was widely publicized and open to anyone from the wider Local Government Area (LGA) of Liverpool to answer. Anyone outside the Liverpool LGA attempting to answer would be locked out of the survey. Thus, the survey was a combination of traditional mail in prompting tools and an online survey. A total of 6284 mail in prompts were sent out and 302 responses were received. Thus, the overall response rate was 4.8%.

Since this response rate is low a number of checks were implemented to test the validity of the survey. Two tests were implemented to test survey validity.

1. **Survey Representativeness**

We tested if the survey sample had a similar distribution as the underlying resident population. The age and sex distribution of the survey population are compared below with the Liverpool LGA population. The survey has an under-representation of 0-39 year old males and has an over-representation of 40-59 year old females (Table 1, below).

:

Table S1: Percentage distributions by Age and Sex in the survey compared to Liverpool LGA

	**Age**	**0-39 Years**	**40-59 Years**	**60-79 Years**
**Survey *(n=239)**	**Male**	10	15	3
	**Female**	32	33	7
	**Age**	**0-39 Years**	**40-59 Years**	**60-79 Years**
**Subset used in analysis (n=136)**	**Male**	10	12	2
	**Female**	34	35	7
	**Age**	**0-39 Years**	**40-59 Years**	**60-79 Years**
**Liverpool LGA (census 2016)**	**Male**	30	13	6
	**Female**	30	14	7

Table S2: Comparing early responders with late responders

The first respondent responded on 14th March 2017, while the last respondent answered the survey on 12th April 2017. We thus split the respondents into two groups, March and April respondents and tabulated them by age and sex. The two groups were quite similar, the only difference being somewhat more females in the 40-59 age group in April compared to, in March. If the age-sex distributions in the two months are compared, the differences were not statistically significant. Difference between male respondents: Chi-Squared Statistic 0.1091, p value 0.95; difference between female respondents, Chi-Squared Statistic 2.045, p value 0.36.

March Respondents			April Respondents		
**Age group**	**Male**	**Female**	**Age group**	**Male**	**Female**
**0-39 Years**	11.96	29.35	**0-39 Years**	8.24	35.29
**40-59 Years**	18.48	28.26	**40-59 Years**	10.59	38.82
**60-79 Years**	3.26	8.70	**60-79 Years**	2.35	4.71

Table S3: Correlations (Kendall's Tau) between different objective greenspace metrics

**100 meter buffer**			
	Mean NDVI	*Percent Parks*	*Percent Tree Canopy*
Mean NDVI		0.09	0.38
Percent Parks			0.07
Percent Tree Canopy			
**10 min walking buffer**			
	Mean NDVI	*Percent Parks*	*Percent Tree Canopy*
Mean NDVI		0.10	0.30
Percent Parks			0.07
Percent Tree Canopy			

Table S4

**Logistic Regression Model Used**	**Confounders**
$logit(Y) = \beta_0 + \alpha + \sum_{i=1}^{k} \beta_i$<br><br>Where Y is the odds of agreeing or strongly agreeing with the statement "There is tree cover or canopy along the footpaths in my local area" in models 1-6 and with the statement "There is lots of greenery (trees, bushes, gardens) around my local area" in models 7-12.<br><br>β_0 is the intercept of the model, or the expected log odds of agreement or strong agreement when all the predictors are set to zero.<br><br>α is the objective greenspace/greenness variable being estimated.<br><br>β_is are the set of confounders adjusted for in the models. There are k possible predictors, with k being equal to 5 in all odd numbered models (1, 3, 9 etc.), and being equal to 7 in all even numbered models (2, 4, 12 etc.); also see confounders in the right column. For a list of cofounders, see the column to the right.	The models included the following confounders in addition to the objective greenspace related variable:<br><br>Confounders in Models 1,3,5,7,9,11<br><br>1. Age (Categorized into three age groups; 0 to 39 years, 40 to 59 years and 60 to 79)<br>2. Sex (Male, Female)<br>3. Country of birth (Australian born, Overseas born)<br>4. Education (Vocational certificate or diploma and university or other tertiary institute degree or higher, versus high school or less)
	The models included the following confounders in addition to the objective greenspace related variable:<br><br>Confounders in Models 2,4,6,8,10,12<br><br>1. Age (Categorized into three age groups; 0 to 39 years, 40 to 59 years and 60 to 79)<br>2. Sex (Male, Female)<br>3. Country of birth (Australian born, Overseas born)<br>4. Education (Vocational certificate or diploma and university or other tertiary institute degree or higher, versus high school or less)<br>5. Recreational walking (dichotomized at the median of twice a week)<br>6. Utilitarian walking dichotomized at the median of 60mins/week
